# Missouri Valley

**Published:** 1899-11

**Authors:** James Kelly

**Affiliations:** Secretary


### SOCIETY PROCEEDINGS.

### MISSOURI VALLEY VETERINARY MEDICAL ASSOCIATION.

THE twenty-first regular meeting was convened at the Kansas City Veterinary College, 1404 Holmes Street, Kansas City, Mo., Monday evening, October 23d, with the following members and visitors present: Members—Drs. Linscott, Ernst, Wright, Kaupp, Brown, Milnes, Verschelden, Cock, Stewart, Buckley, Netherton, Moore, Sloan, Biart, Bennett, Patterson, and Kelly. Visitors—Drs. Metsker, Neal, Allen, Cooper, Steel, Boyce, Groves, Nixon, Black, and about thirty students.

An interesting and instructive clinic was held at the college, and the following operations were successfully performed: "Ovariotomy per Vagina," by Dr. H. G. Patterson; "Ovariotomy per Flank," by Dr. W. N. Nixon; "Peroneal Tenotomy," by Dr. R. C. Moore; "Neurectomy," by Dr. J. R. Black; "Arytenoideraphy and Median Neurectomy," by Dr. R. C. Moore.

Owing to the absence of the President and Vice-Presidents, Dr. John Ernst, of Leavenworth, was elected President *pro tempore.*

There being no business, the Association proceeded to the consideration of papers presented, as follows: "Actinomycosis," by Dr. J. B. Wright; "Counter-irritants," by Dr. J. A. Sloan; "An Unclassified Disease following Parturition," by Dr. O. Verschelden. These papers were all thoroughly discussed, after which the meeting adjourned, to meet in St. Joseph, Mo., in December.

JAMES KELLY,
Secretary.

